# Comprehensive analysis of NAC transcription factors uncovers their roles during fiber development and stress response in cotton

**DOI:** 10.1186/s12870-018-1367-5

**Published:** 2018-07-24

**Authors:** Heng Sun, Meiling Hu, Jianying Li, Lin Chen, Meng Li, Shuqin Zhang, Xianlong Zhang, Xiyan Yang

**Affiliations:** National Key Laboratory of Crop Genetic Improvement, Huazhong Agricultural University, Wuhan, Hubei 430070 People’s Republic of China

**Keywords:** Cotton, NAC, Evolutionary analysis, Transactivation, Fiber development, Stress response

## Abstract

**Background:**

Transcription factors operate as important switches of transcription networks, and NAC (NAM, ATAF, and CUC) transcription factors are a plant-specific family involved in multiple biological processes. However, this gene family has not been systematically characterized in cotton.

**Results:**

Here we identify a large number of genes with conservative NAC domains in four cotton species, with 147 found in *Gossypium arboreum*, 149 in *G. raimondii*, 267 in *G. barbadense* and 283 in *G. hirsutum*. Predicted membrane-bound *NAC* genes were also identified. Phylogenetic analysis showed that cotton NAC proteins clustered into seven subfamilies and homologous protein pairs showed similar characteristics. Evolutionary property analysis revealed that purifying selection of *NAC* genes occurred between diploid and polyploid cotton species, and variation analysis showed *GhNAC* genes may have been subjected to selection and domestication. NAC proteins showed extensive transactivation and this was dependent on the C-terminus. Some development and stress related cis-elements were enriched in the promoters of *GhNAC* genes. Comprehensive expression analysis indicated that 38 *GhNAC* genes were candidates for involvement in fiber development, and 120 in stress responses. Gene co-expression network analysis revealed relationships between fiber-associated *NAC* genes and secondary cell wall (SCW) biosynthesis genes.

**Conclusions:**

*NAC* genes were identified in diploid and tetraploid cotton, revealing new insights into their evolution, variation and homology relationships. Transcriptome analysis and co-expression network indicated roles for *GhNAC* genes in cotton fiber development and stress response, and NAC genes may prove useful in molecular breeding programmes.

**Electronic supplementary material:**

The online version of this article (10.1186/s12870-018-1367-5) contains supplementary material, which is available to authorized users.

## Background

NAC (NAM, ATAF, and CUC) proteins are among the largest plant-specific transcription factor (TF) superfamily with 117 members in *Arabidopsis*, 151 in rice, 152 in soybean, and 251 in switchgrass [1–3]. In general, the structures of NAC proteins can be divided into two regions, the conserved N-terminal DNA-binding domains (BD) and the high divergent C-terminal transcriptional regulatory regions (TR) [4, 5]. The BD contains about 150~ 160 amino acid residues and it can be further classified into five subdomains (A-E), associated with DNA binding, formation of homodimers or heterodimers and nuclear localization. The TRs are required to activate/repress transcription, and the high divergence amongst family members accounts for the diverse functions of NAC proteins [5]. Some NAC proteins have a transmembrane (TM) motif in the C-terminus, and show subcellular localization at the plasma membrane or endoplasmic reticulum; these proteins may be regulated by post-translational regulation under specific conditions [6–8]. Several species have membrane-bound NAC transcription factors, with 13 in *Arabidopsis*, 13 in tomato, 11 in soybean and 5 in rice, and most are induced by abiotic stresses [1, 9–12].

Previous studies showed that NAC proteins are widely implicated in the regulation of transcriptional reprogramming associated with diverse developmental processes [13–16]. Mutation of *NAM*, the firstly identified *NAC* gene, failed to develop a shoot apical meristem (SAM) in *Petunia* embryos [17]. *Arabidopsis* CUC1 and CUC2 showed a similar function in SAM formation [18]. In addition, NAC transcription factors play an important role in secondary cell wall (SCW) biosynthesis in plants [13, 19, 20]. Secondary wall biosynthesis-related NAC transcription factors, including VASCULAR-RELATED NAC-DOMAIN (VND) and NAC SECONDARY WALL THICKENING PROMOTING FACTOR1 (NST) are transcriptional switches that regulate SCW biosynthesis in *Arabidopsis* [13]. Seven *VND* genes (*VND1–7*) were identified by an in vitro xylem vessel element inducible system, and microarray and promoter analysis revealed that these genes are up-regulated during in-vitro xylem vessel element formation and are specifically expressed in vascular cells [21]. Overexpressing *VND* genes up-regulated the expression of secondary wall-associated genes and showed thickened secondary walls [21, 22]. The *NST* genes (*NST1–3*) are preferentially expressed in xylary and extraxylary fibers, and regulate the formation of the SCW [13, 23]. The function of secondary wall-related *NAC* genes may be well conserved across the plant kingdom [13, 24, 25].

Plants are constantly confronted with a variety of abiotic stresses, such as drought and high salinity, and these stress conditions adversely affect plant growth, with a severe impact on agricultural productivity. To date, most evidence suggests that NAC proteins play roles in abiotic stress response. Many stress-responsive NAC proteins are positive regulators of abiotic stress tolerance, though some are negative regulators [5, 16, 26]. In *Arabidopsis*, AtRD26 (ANAC72) functions as a transcriptional activator in abscisic acid (ABA) mediated stress signaling [27]. Transgenic plants overexpressing *ANAC019*, *ANAC055* or *ANAC072* show enhanced drought tolerance by inducing several stress-inducible genes [28]. However, NAC016 negatively regulates drought tolerance by binding to the *NAC016-specific binding motif* (*NAC16BM*) in the *AREB1* promoter and repressing the expression of *AtAREB1*. Meanwhile, NAP, encoded by a NAC016 target gene, binds to the promoter of *AREB1* and suppresses its transcription [29]. Some stress-related *NAC* genes have been identified in rice and have potential for improving stress tolerance. Rice plants overexpressing *STRESS-RESPONSIVE NAC1* (*SNAC1*) showed significantly improved drought and salt tolerance [30]. A stress-responsive *NAC* gene, *SNAC3* positively regulates drought and heat tolerance in rice by modulating ROS homeostasis [31].

Cotton is one of the most important economic crops, and NAC proteins play important roles in cotton fiber development and stress tolerance. The process of fiber development can be divided into four continuous stages: initiation, elongation, secondary cell wall deposition and maturation [32]. The cotton NAC transcription factor, *GhFSN1*, was recently identified as a regulator of fiber SCW formation [25]. *GhFSN1* is expressed in fibers during the SCW thickening stage, and transgenic experiments indicate that *GhFSN1* positively regulates SCW thickness but negatively regulates fiber length. *GhFSN1* directly regulates the expression of secondary cell wall-related genes in cotton. Other NAC genes have been identified as involved in stress tolerance in cotton. *GhNAC79* with relatively higher transcript accumulation at later stages of cotyledon and fiber development, overexpression of *GhNAC79* showed improved drought tolerance in both cotton and *Arabidopsis* [33]. A NAC transcription factor gene, *GhATAF1* has been reported to play a role in crosstalk between biotic and abiotic stress responses [34]. Cotton plants overexpressing *GhATAF1* show improved salt stress tolerance, but also an increased susceptibility to the fungal pathogens *Verticillium dahliae* and *Botrytis cinerea*. Previous genetic studies identified stress-responsive *NAC* genes in cotton. *GhNAC1* - *GhNAC6* were identified by sequence homology, and these genes are differentially regulated under abiotic stress [35]. Seven *GhNAC* genes, *GhNAC7* - *GhNAC13,* which are preferentially expressed in roots, have been shown to be involved in stress responses [36]. The expression patterns of 60 *GhNAC* genes, which were located on D sub-genome, were analyzed during stresses and phytohormones [37]. We speculate that NAC proteins show functional conservation in SCW formation and stress response in plants. Although a few *NAC* genes involved in fiber development and stress response have been characterized in cotton, the regulatory networks of *NAC* genes are still poorly understood.

Genome-wide analysis of *NAC* genes allows gene functional studies. The genome data for two diploid cotton species (*G. arboreum*, *G. raimondii*) and two tetraploid species (*G. barbadense*, *G. hirsutum*), and public transcriptome data sets have made possible the genome-wide identification and analysis of the gene families in cotton [38–42]. However, the *NAC* gene family is poorly characterized, especially in Upland cotton (*G. hirsutum*), which accounts for more than 90% of cultivated cotton worldwide; although such an analysis has been performed in diploid cotton [43, 44]. In the present study, the *NAC* gene family was comprehensively studied in four cotton species, to reveal phylogenetic relationships, synteny, genetic variation, co-expression networks, transactivation and cis-element analysis. We also linked *NAC* gene family members to fiber development and stress responses.

## Results

### Genome-wide identification of *NAC* family genes in *Gossypium spp*

**Fig. 1:**
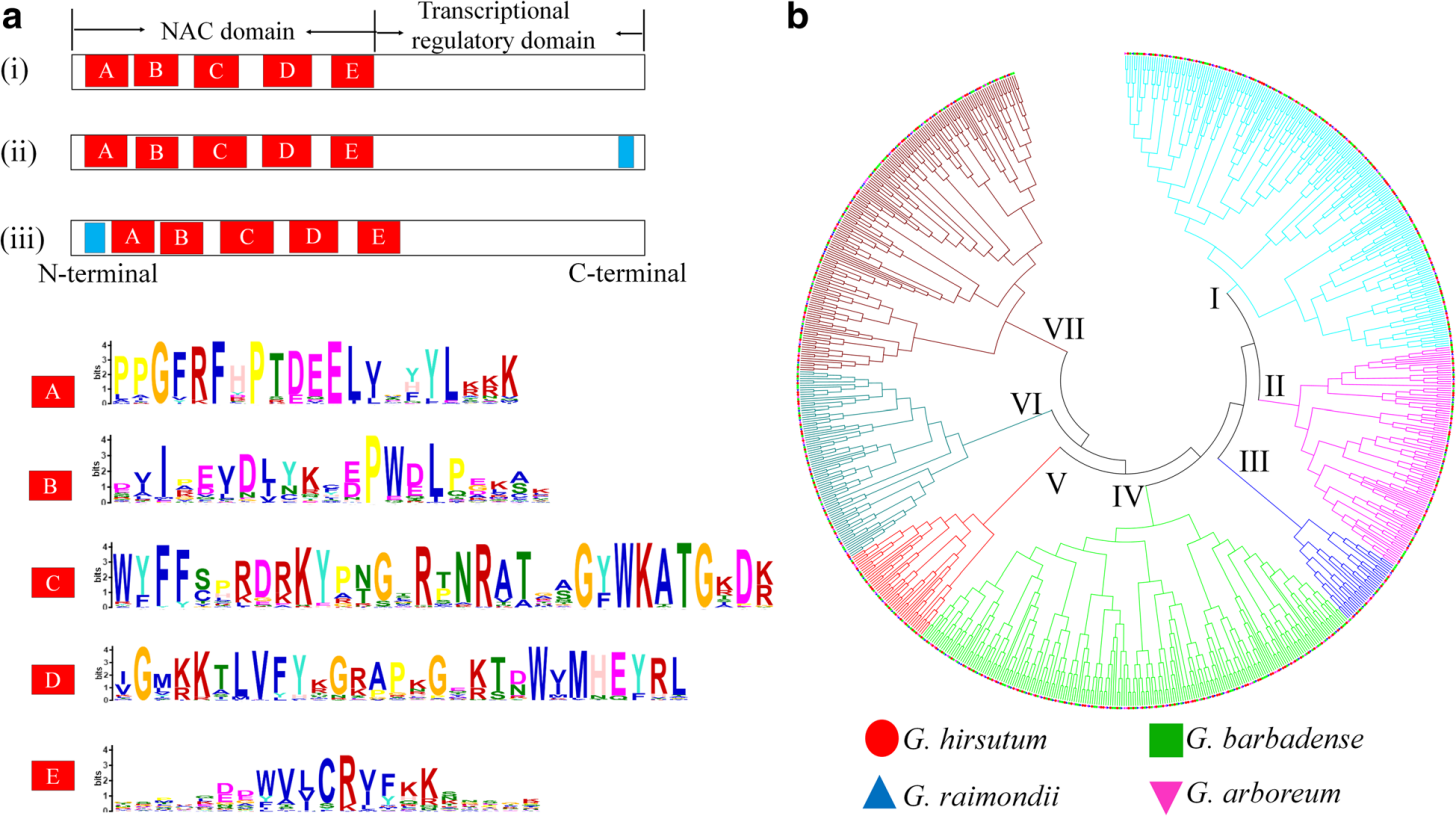
The conserved domain and phylogenetic analysis of NAC proteins in cotton. **a** Schematic representation of NAC proteins. (i) Typical NAC protein with a highly conserved NAC domain (A-E subdomains) at the N-terminal; (ii) Membrane-bound NAC transcription factors with a transmembrane motif (TM, blue square) at the C-terminal; (iii) Membrane-bound NAC transcription factors with a transmembrane motif (TM, blue square) at the N-terminal. **b** Phylogenetic tree of NAC proteins in four cotton species. *G. hirsutum* (red circle), *G. barbadense* (green square), *G. raimondii* (blue triangle), *G. arboreum* (magenta triangle)

Based on the PlantTFDB database and multiple sequence alignment analysis, we identified multiple non-redundant and complete *NAC* genes in four cotton species, with 147 found in *Gossypium arboreum*, 149 in *G. raimondii*, 267 in *G. barbadense* and 283 in *G. hirsutum* (Additional file 1: Table S1). The predicted cotton NAC protein sequence lengths, molecular weights and protein isoelectric points (pI) were analyzed (Additional file 2: Figure S1, Additional file 3: Table S2). For example, the full length of GhNAC proteins range from 168 to 1219 amino acids, with molecular weights ranging from 20.1–135.6 kDa, and isoelectric points from 4.39 to 9.8. Multiple sequence alignment and conservative motif analysis showed that cotton NAC proteins possess a conserved NAC domain, which includes five subdomains (A-E), with some conserved amino acid present (Fig. 1a). A phylogenetic tree was constructed to gain insights into the evolutionary relationships between NAC proteins in *Gossypium*. NAC proteins from four cotton species group into seven subfamilies (I-VII), and each subfamily contains the NAC proteins derived from four cotton species (Fig. 1b).

There are 13, 12, 21 and 22 predicted membrane-bound NAC proteins were identified in four cotton species respectively (Red color in Additional file 1: Table S1). Most of the predicted membrane-bound NAC proteins had one TM at the C terminal, such as GhNAC157 (Additional file 4: Figure S2a). GaNAC111 possesses two predicted TM at the C terminal, and eight NAC proteins (GaNAC68, GaNAC57, GrNAC79, GbNAC23, GbNAC104, GbNAC184, GhNAC258, GhNAC169) have one TM preceding the conserved NAC domain (Additional file 4: Figure S2b, c). The predicted membrane-bound NAC proteins are mainly distributed in subfamilies II and III (46 out of 68), with none in subfamily VI. Phylogenetic analysis of these NAC proteins compared with the membrane-bound NAC proteins in *Arabidopsis*, tomato and rice show that some clade genes are present in diverse species (Additional file 4: Figure S2d).

**Fig. 2:**
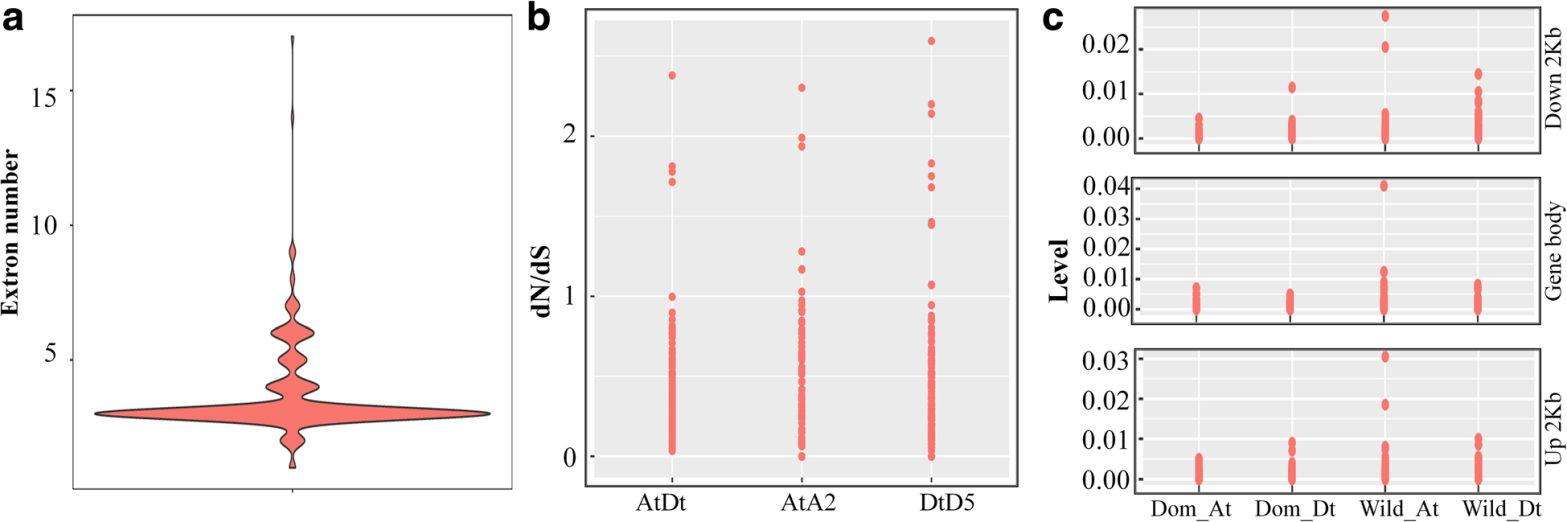
Evolutionary analysis of NAC proteins in cotton. **a** Statistical analysis of exon numbers of *NAC* genes in *G. hirsutum*. **b** The ratio of non-synonymous to synonymous substitutions (d_N_/d_S_) of *NAC* genes in inter-genomic (At Dt) and intra-genomic (A2 At and D5 Dt). **c** The SNP density of *GhNAC* genes in Upland cotton populations. The gene body, 2 kb upstream and 2 kb downstream sequences of each *GhNAC* gene were selected for analysis

Gene structure analysis based on exon/intron organization is an important and helpful method for studying genetic evolution. Most *GhNAC* genes had three exons (193/283, 68.2%) (Fig. 2a), though the homologous gene pair *GhNAC111* and *GhNAC253* contained only one exon, and *GhNAC4* possessed the highest number with seventeen exons. *GhNAC* genes in the same subfamily showed conserved structural similarities, and almost all of the most closely related gene pairs shared the same structural features.

### Genome synteny and variation analysis of NAC genes

Genome synteny analysis is important for deciphering a genome’s evolutionary history. We examined the distribution and gene homology of *NAC* genes by a genome synteny analysis (Additional file 5: Figure S3). For gene loci analysis, we found all chromosomes possessed at least one *NAC* gene in At (A subgenome, with lower-case ‘t’ denoting tetraploid) and Dt. In *G. hirsutum*, more *NAC* genes are distributed on chromosomes A05, A11, D05 and D11 than on other chromosomes, while 25 *GhNAC* genes have no distinct chromosome location and could not be accurately mapped (Additional file 6: Table S3). We further examined homologous gene pairs by multiple homologous comparison analysis, and our results showed that there are 114, 138, 136 homologous gene pairs were identified from At to Dt, At to A2, Dt to D5, respectively (Additional file 7: Table S4). Furthermore, we found homologous genes in different cotton species show close evolutionary relationships and gene characteristics, such as gene structure and predicted protein molecular weight.

The non-synonymous (d_N_) and synonymous (d_S_) substitution rates within and between cotton species were calculated to explore evolutionary dynamics and selection pressures. Most of the d_N_ is less than d_S_ (d_N_/d_S_ < 1) in the inter-genomic (At and Dt) and intra-genomic (A2 and At or D5 and Dt) comparisons, suggesting that purifying selection of *NAC* genes has occurred between both diploid and polyploid cotton species (Fig. 2b).

Upland cotton is the main cultivated cotton worldwide, and natural selection and domestication have played important roles in its development. Single nucleotide polymorphism (SNP) density in the gene body, and the regions 2 kb upstream and 2 kb downstream of *GhNAC* genes were analyzed based on the genome data for 31 wild cotton and 321 domesticated Upland cotton accessions. As expected, the SNP density of these three regions in wild cotton was higher than that of cultivated cotton, indicating that the evolution of cultivated cotton experienced selection and domestication. Moreover, the variant range of A subgenome was higher than that of the D subgenome in wild cotton, while the SNP density of D subgenome was higher than that of the A subgenome in the 2 kb upstream and 2 kb downstream regions in cultivated cotton (Fig. 2c).

### Cis-element analysis of *NAC* genes in *G. hirsutum*

**Fig. 3:**
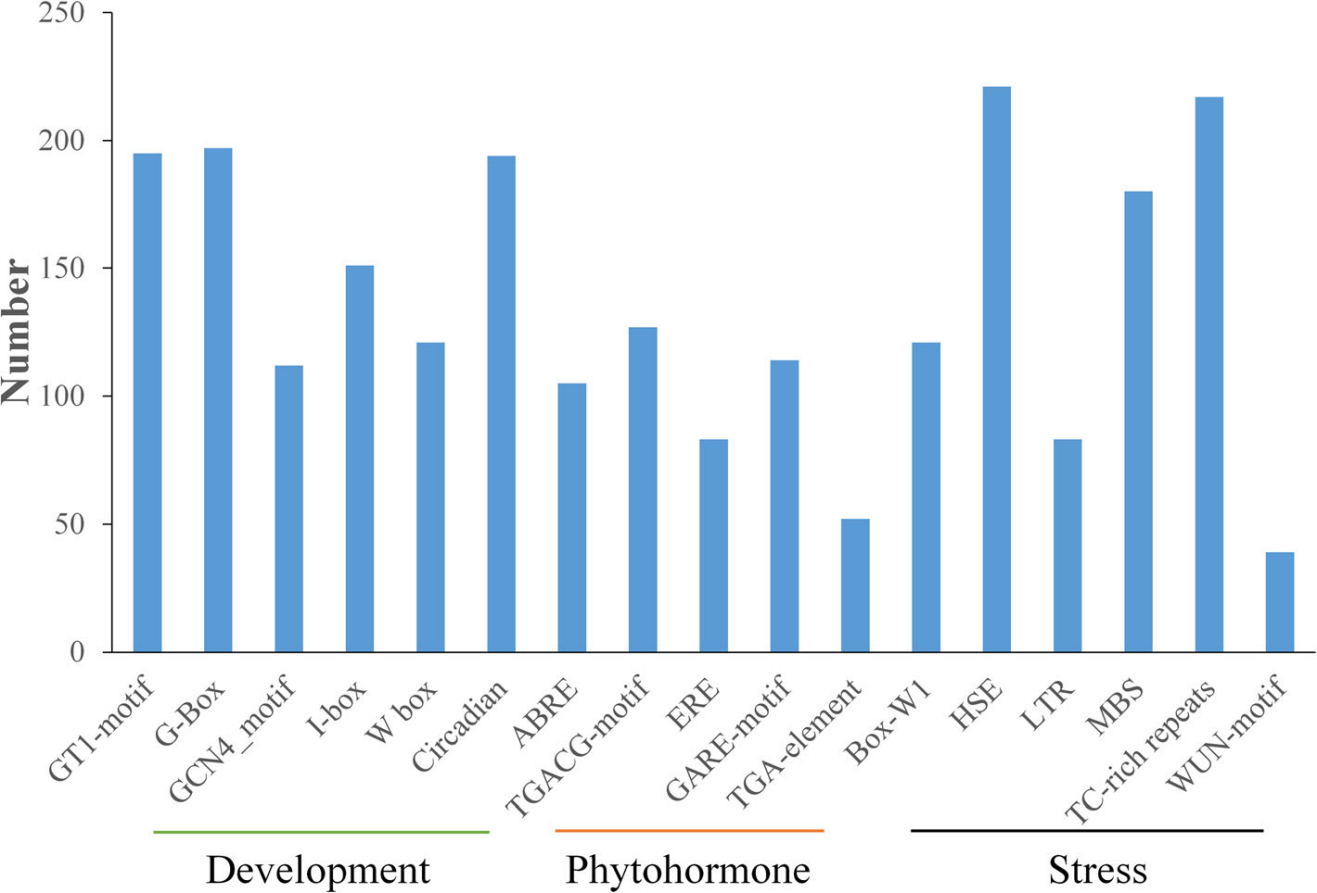
Cis-element analysis of *NAC* genes in *G. hirsutum*. Cis-element analysis of *GhNAC* gene promoter. The 1.5-kb upstream sequence of the start codon of each NAC gene were analyzed by the PLANTCARE database

Promoter analysis is an effective method to study potential transcriptional regulation of genes. There are abundant regulatory elements existing in the promoter region of *GhNAC* genes, which are predicted to be involved in phytohormone responses, development and stress responses (Fig. 3, Additional file 8: Table S5). The 5 most commonly found cis-elements include the heat stress response element (HSE, 221 out of 283, 78.1%), TC-rich repeats (76.7%), G-box (69.6%), GT1-motif (68.9%), and circadian (68.6%). HSE and TC-rich repeats are involved in defense and stress responsiveness, while circadian and GT1-motif are involved in light response and development. Our results implied that *GhNAC* genes might play an important role in both stress response and development in cotton.

### Identification of fiber-associated NAC family members in *G. hirsutum*

**Fig. 4:**
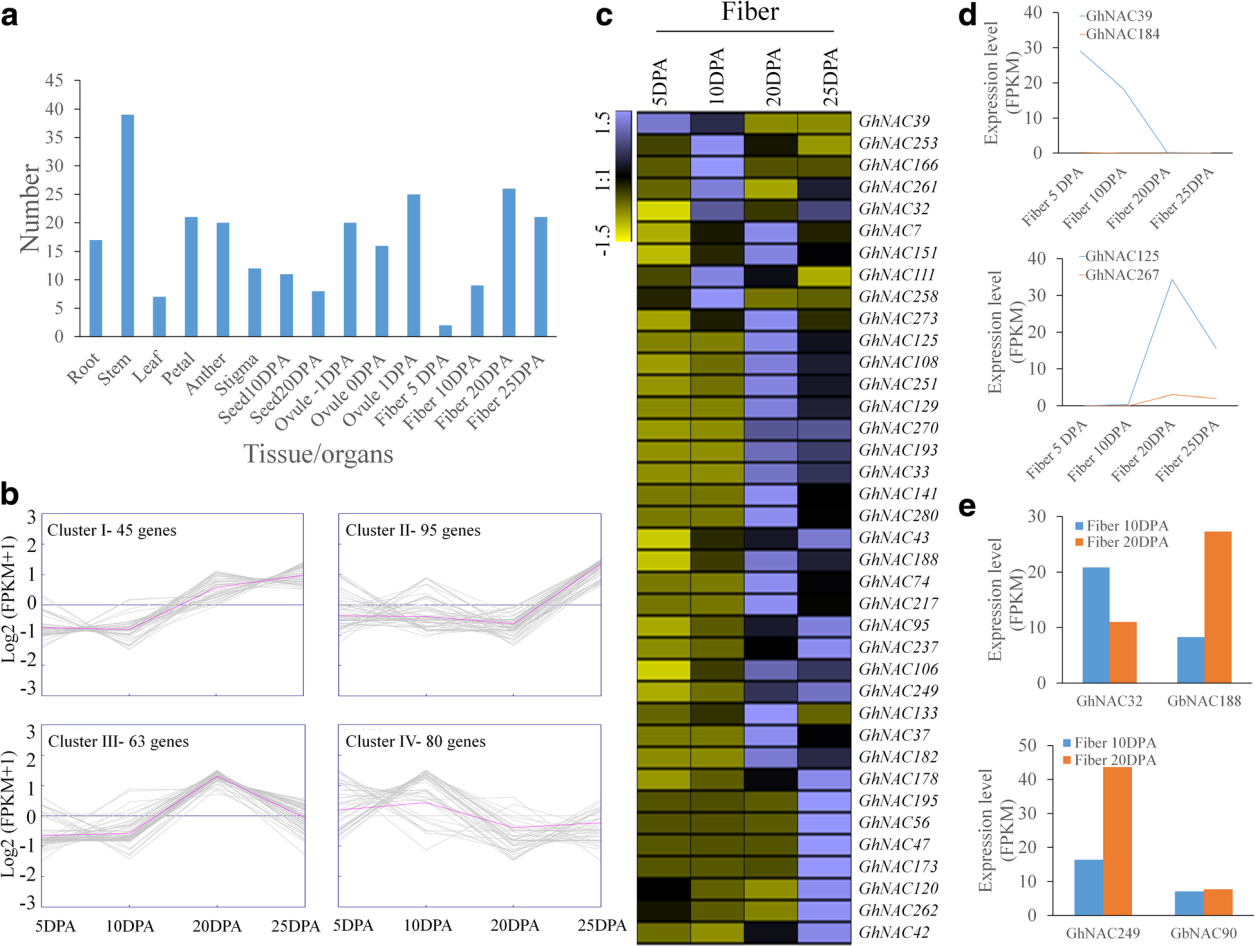
Expression analysis of *GhNAC* genes during fiber development. **a** The number of highly expressed *NAC* genes (FPKM ≥20) in different tissues/organs. The expression of *NAC* family members in different tissues/organs were investigated using transcriptome datasets of *G. hirsutum*. **b** Cluster analysis of the *GhNAC* expression patterns in fiber development. The grey lines indicate the expression levels of genes. **c** Heat-map of 38 highly and differently expressed *NAC* genes in fiber development based on transcriptome datasets. The expression values (FPKM) were normalized using Genesis. **d** The bias expression of fiber related *NAC* genes in *G. hirsutum*. **e** Two gene pairs showed discordant expression changes between *G. hirsutum* and *G. barbadense*

The available and public transcriptome data sets have made it possible to comprehensively analyze *NAC* genes expressed during development processes in cotton. We collected the transcriptome data sets from different tissues/organs, including the root, stem, leaf, petal, anther, stigma, ovule, seed and fiber (Additional file 9: Figure S4a) and identified 198 (70%) *GhNAC* genes expressed at least in one tissue, and 37 (13.1%) genes were expressed in all tissues (FPKM ≥1). Some *GhNAC* genes were highly expressed (FPKM ≥20) in stem, ovule and fiber (Fig. 4a).

Cotton fibers are the most important natural textile materials worldwide. The transcriptome data were analyzed during fiber development over four developmental stages (5 DPA, 10 DPA, 20 DPA and 25 DPA). There are 142 (50.2%) *GhNAC* genes identified as being expressed during fiber development. The expression patterns of all *GhNAC* genes during fiber development were divided into four subclusters (cluster I-IV) (Fig. 4b). *GhNAC* genes belonging to subclusters I-III showed expression changes during fiber development. Genes from cluster I were highly expressed at 20 and 25 DPA, genes from cluster II showed low expression levels at early stages of development and then up-regulated at 25 DPA, while genes from cluster III were up-regulated at 20 DPA and then down-regulated at 25 DPA. Genes from cluster IV showed a high expression levels during 10 DPA and then low expression levels subsequently.

We identified 38 highly expressed (FPKM ≥20) *GhNAC* genes with obvious expression changes during fiber development, and these genes are evenly distributed in the A and D subgenomes (Fig. 4c, Additional file 10: Table S6). There are 13 homologous pairs within these genes, and two genes of each homologous gene pair showed similar expression trends. We found ten genes (*GhNAC33*, *GhNAC39*, *GhNAC108*, *GhNAC125*, *GhNAC166*, *GhNAC193*, *GhNAC217*, *GhNAC251*, *GhNAC273*, *GhNAC280*) that showed preferential expression in fibers compare with other tissues or organs. Two genes (*GhNAC7*, *GhNAC151*) showed high expression at the elongation stage and SCW thickening stage (10–25 DPA), 12 genes (*GhNAC33*, *GhNAC43*, *GhNAC74*, *GhNAC95*, *GhNAC106*, *GhNAC129*, *GhNAC188*, *GhNAC193*, *GhNAC217*, *GhNAC237*, *GhNAC249*, *GhNAC270*) showed high expression at the transition and SCW thickening stage (20–25 DPA), eight genes (*GhNAC42*, *GhNAC47*, *GhNAC56*, *GhNAC120*, *GhNAC173*, *GhNAC178*, *GhNAC195*, *GhNAC262*) showed high expression specifically at the SCW thickening stage (25 DPA) and nine genes (*GhNAC37*, *GhNAC108*, *GhNAC125*, *GhNAC133*, *GhNAC141*, *GhNAC182*, *GhNAC251*, *GhNAC273*, *GhNAC280*) showed specific high expression at the transition stage. Three genes (*GhNAC125*, *GhNAC166*, *GhNAC273*) have high homology with *Arabidopsis NST1*, which may regulate the formation of SCW [20].

Previous reports showed some genes in the At and Dt subgenomes demonstrated biased expression patterns. Of the 38 genes, we found the expression of some *GhNAC* homoeologous genes demonstrated biased expression patterns. *GhNAC39* and *GhNAC125* located in the At subgenome, have preferential expression in fibers, whereas the expression levels of the Dt homoeologous genes were nearly undetectable (Fig. 4d). We investigated the homologous gene expression patterns for the 38 *GhNAC* genes in three other cotton species, using transcriptome data sets for fiber development (10DPA and 20DPA) (Additional file 9: Figure S4b). Most of the genes showed similar expression changes, suggesting the conservation of functions of *NAC* genes in regulating fiber development between diploid and allotetraploid cotton. We also identified two gene pairs (*GhNAC32* and *GbNAC188*, *GhNAC249* and *GbNAC90*) that showed significantly different expression patterns between Upland cotton *G. hirsutum* and extra-long staple cotton *G. barbadense* (Fig. 4e).

**Fig. 5:**
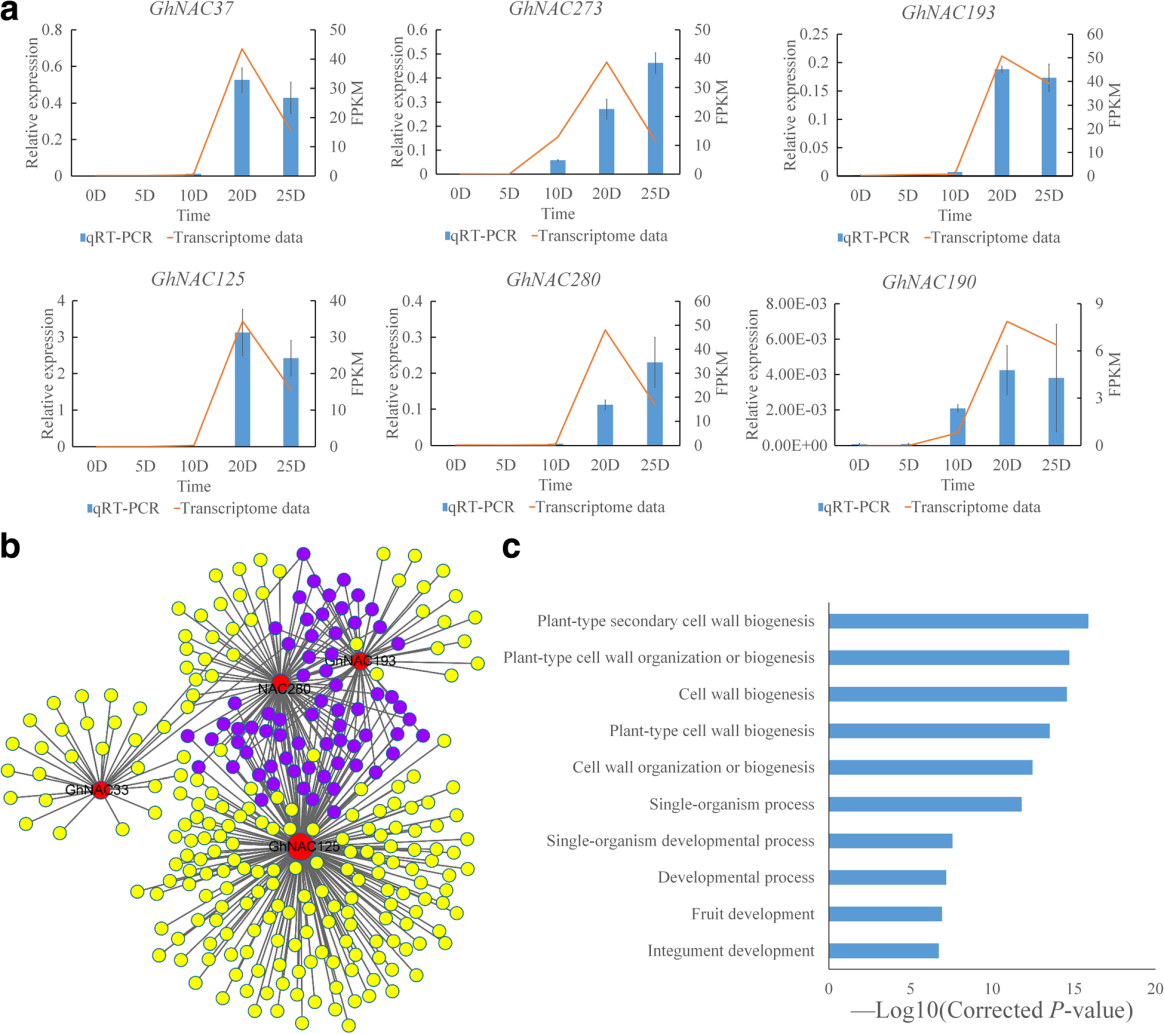
The qRT-PCR and co-expression network analysis of fiber-related *GhNAC* genes. **a** Expression analysis of the selected *GhNAC* genes in fiber development by qRT-PCR. The *GhUBQ7* (GenBank accession number: DQ116441) was used as the internal control to calculate and normalize the expression levels. Bars represent means ± standard error (*n* = 3). The orange lines represent the transcriptome data (FPKM). **b** The co-expression network with gene expression view of *GhNAC* genes in fiber development. The gray lines between two nodes indicate co-expression relationships. The purple solid circles represent overlapped genes. **c** GO enrichment analysis of co-expression genes with *GhNAC33*, *GhNAC125*, *GhNAC193*, *GhNAC280*

Six *GhNAC* genes were selected to verify the expression changes during fiber development progress (0, 5, 10, 20, 25 DPA) by quantitative reverse-transcription-PCR (qRT-PCR) (Fig. 5a). All the selected genes showed almost the similar expression changes compared with transcriptome data sets. The expression of five highly expressed genes (*GhNAC37*, *GhNAC125*, *GhNAC193*, *GhNAC273*, *GhNAC280*), which were selected according to transcriptome data sets, showed sharp increases at the SCW thickening stage.

Four fiber development related *NAC* genes (*GhNAC33*, *GhNAC125*, *GhNAC193*, *GhNAC280*) were selected for co-expression analysis. Two hundred and ninety-one co-expressed genes were identified and some showed overlap with these four genes (Fig. 5b, Additional file 11: Table S7). Gene ontology analysis of the co-expressed genes showed that plant cell wall biogenesis was the most abundant functional term, and we identified cellulose synthase family genes with co-expression relationships, such as *GhCESA7* (Gh_D07G0380), *GhCESA4* (Gh_A07G1871), *GhCESA8* (Gh_D10G0333). This suggests that *NAC* genes might be involved in fiber development by regulating secondary cell wall synthesis (Fig. 5c).

### Identification of stress-related *NAC* genes in *G. hirsutum*

**Fig. 6:**
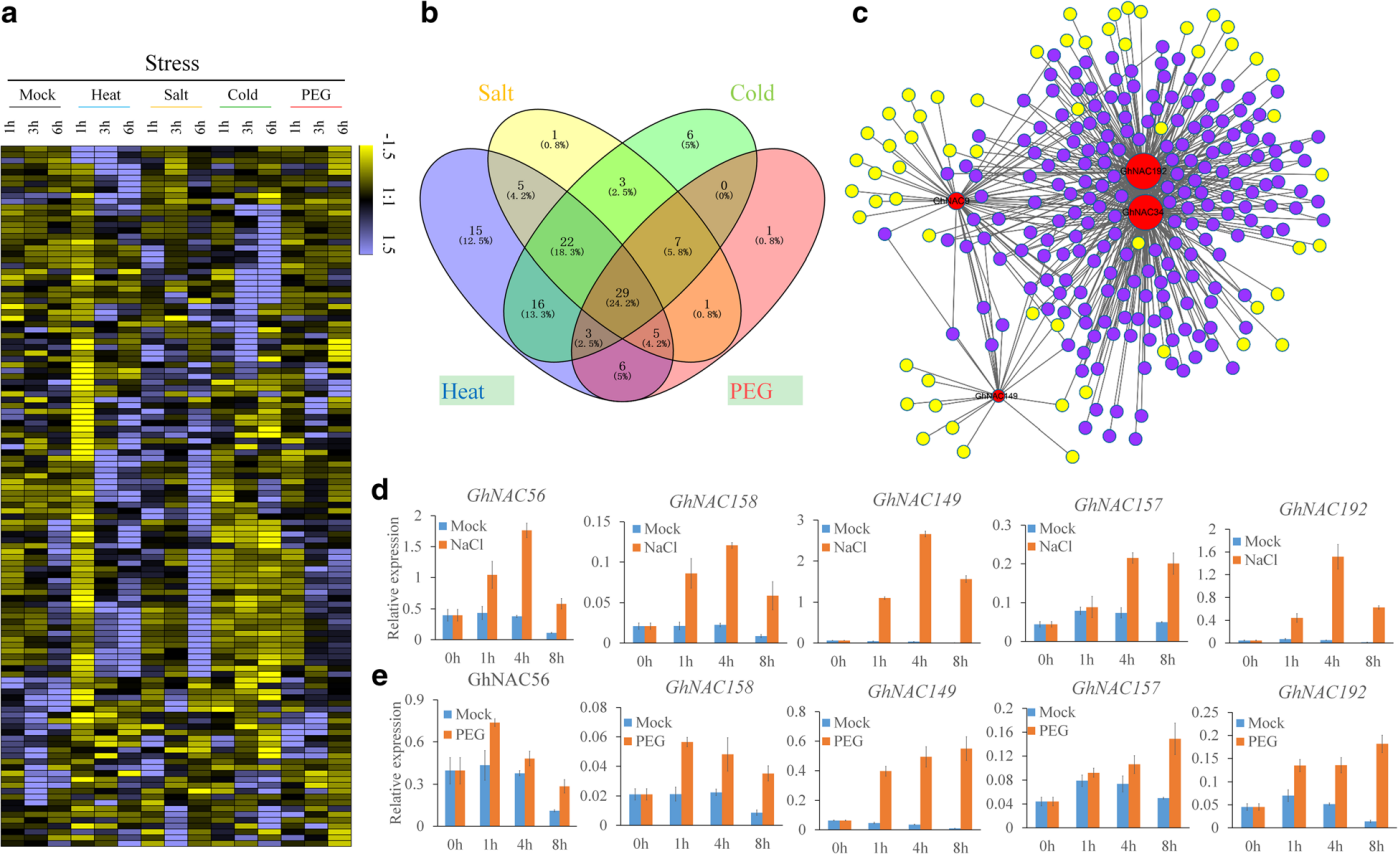
Expression and co-expression network analysis of *GhNAC* genes under abiotic stress. **a** The expression patterns of 120 stress-responsive *GhNAC* genes under abiotic stress. **b** Venn diagram showing overlap of different stress-responsive *GhNAC* genes. **c** The co-expression network of *GhNAC* genes with DEGs in the abiotic stress treatment. The gray lines between two nodes indicate co-expression relationship. The purple solid circles represent overlapped genes. **d** The expression of selected *GhNAC* genes under salt stress (200 mM NaCl) in leaves by qRT-PCR. **e** The expression of selected *GhNAC* genes under osmotic stress (15% PEG) in leaves by qRT-PCR. The *GhUBQ7* (GenBank accession number: DQ116441) was used as the internal control. Bars represent means ± standard error (*n* = 3)

*NAC* genes have received much attention as important regulators in various stress signaling pathways. We used the public transcriptome data sets of *G. hirsutum* treated with high temperature (heat), salinity, cold and polyethylene glycol (PEG) to investigate the expression patterns of *GhNAC* genes under abiotic stress (Additional file 12: Figure S5a). One hundred and twenty-four (43.8%) *GhNAC* genes expressed at least in one stress (FPKM ≥1) and 120 (42.4%) of these genes with two-fold different expression compared with controls in at least one treatment (Fig. 6a, Additional file 10: Table S6). There were 101, 73, 86 and 52 differentially expressed *GhNAC* genes were identified in heat, salinity, cold and PEG stress treatments, respectively. Comparative analysis showed that 29 *GhNAC* genes exhibited overlapping differential expression under the four stress conditions (Fig. 6b). Heat and cold had the most overlapping genes (70 genes), whereas cold and PEG stress had the least (39 genes). We also identified 10 stress-related *GhNAC* genes (*GhNAC9*, *GhNAC15*, *GhNAC34*, *GhNAC47*, *GhNAC56*, *GhNAC149*, *GhNAC158*, *GhNAC173*, *GhNAC192*, *GhNAC195*) that are homologous with stress-related *NAC* genes in *Arabidopsis* and rice, such as *RD26*, *ANAC019*, *SNAC1* and *SNAC2*, which are reported to be involved in multiple stress responses (Additional file 12: Figure S5b). The expression patterns were closely related to evolutionary relationships. Our results indicate that some *GhNAC* genes might play conserved functions in stress responses in plants.

Four stress-related *GhNAC* genes (*GhNAC9*, *GhNAC34*, *GhNAC149*, *GhNAC192*) were selected for co-expressed network analysis. There were 58, 207, 22 and 227 co-expressed differentially expressed genes (DEGs) detected in *GhNAC9*, *GhNAC34*, *GhNAC149* and *GhNAC192*, respectively (Fig. 6c, Additional file 11: Table S7). Some co-expression genes were associated with stress responses, such as late embryogenesis abundant protein genes (Gh_D10G0248, Gh_D11G0978, Gh_D11G2003), heat shock transcription factor genes (Gh_D05G0228, Gh_D05G0307), peroxidase superfamily protein genes (Gh_D04G0130, Gh_A09G1575, Gh_A10G1317) and phytohormone responsive genes (Gh_A05G0278, Gh_A05G0782, Gh_A05G3589). The 267 specific co-expression genes were annotated based on functional gene ontology using KOBAS 3.0, and some major enrichment categories were identified: response to acid chemical, response to endogenous stimulus, response to water deprivation (Additional file 12: Figure S5c).

The expression profiles of some stress-related *GhNAC* genes were verified by qRT-PCR under 200 mM NaCl, simulating salt stress; and 15% PEG, simulating osmotic stress (Fig. 6d, e). The qRT-PCR results are consistent with transcriptome data sets, showing that the selected *GhNAC* genes are significantly induced by these two stresses. However, it is worth noting that these selected *NAC* genes are barely induced by ABA treatment, with the exception of *GhNAC164* (Additional file 13: Figure S6).

### Transactivation assay of GhNAC proteins

**Fig. 7:**
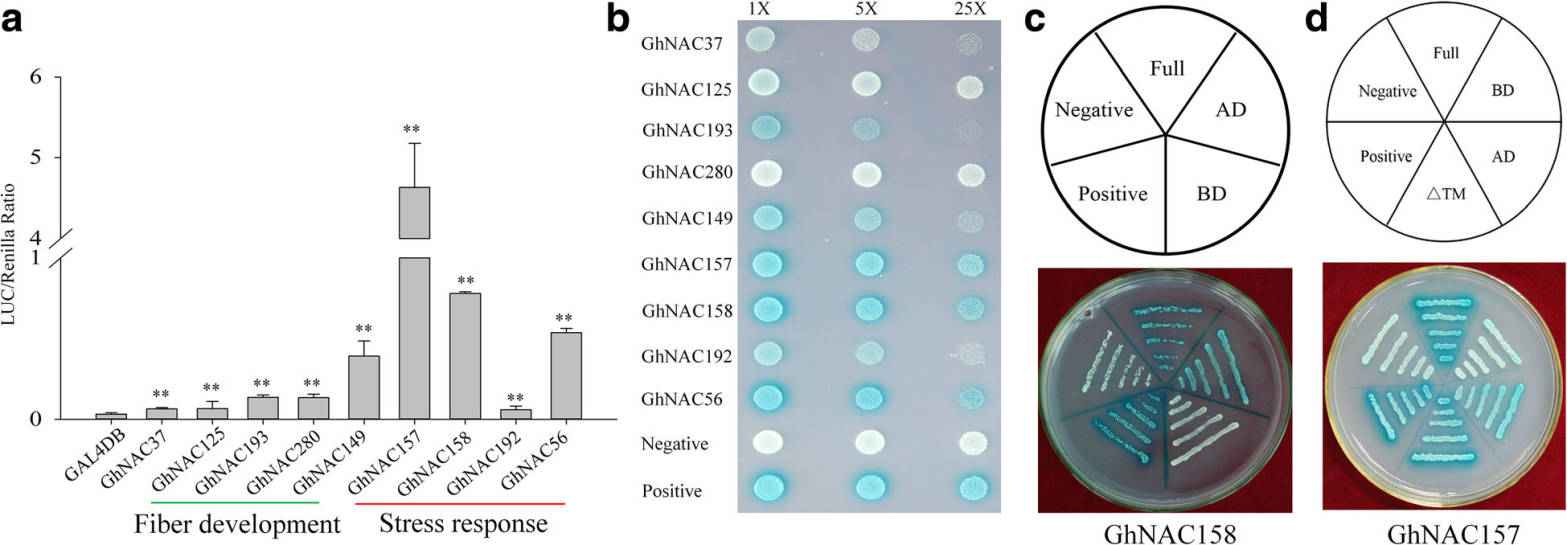
Transactivation analysis of *GhNAC* genes. **a** The transcriptional activity of selected NAC proteins in cotton embryogenic callus protoplasts. Renilla LUC was used as the internal control. Three independent experiments were performed. Asterisks indicate significant differences, ** *P* ≤ 0.01, student’s t-test. The LUC/Renilla Ratio of each gene compared with control (GAL4DB), respectively. **b** Transactivation assay of NAC proteins in yeast strain Y2H. The transactivation activities were determined by the growth on SD medium without Tryptophane (Trp) and added X-α-Gal (SD-Trp + X-α-Gal). The photograph was taken after 36 h incubation. Positive control: pGBKT7–53; negative control: pGBKT7-Lam. **c** Transactivation assay of GhNAC158. Full: the full length of GhNAC158, BD: NAC domain (Binding Domain) of GhNAC158, AD: the C terminal (Activation Domain) of GhNAC158. **d** Transactivation assay of GhNAC157. △TM: the transmembrane motif (TM) deleted form of GhNAC157

Transcriptional activation is an important characteristic of NAC transcription factors. To investigate the transactivation capacity of NAC proteins in cotton, we performed a protoplast transient expression system, which based on the interaction between the DNA binding domain of GAL4 (GAL4DB) and the binding sites of GAL4(5X)-TATA-LUC. The full-length cDNA of selected *GhNAC* genes (stress related: *GhNAC56*, *GhNAC149*, *GhNAC157*, *GhNAC158*, *GhNAC192*; fiber development related: *GhNAC37*, *GhNAC125*, *GhNAC193*, *GhNAC280*) were cloned into the vector GAL4DB respectively. Results showed that in all cases the luciferase (LUC)/Renilla LUC ratio of GAL4DB-GhNACs was to a greater extent higher than that of control GAL4DB, and the membrane-bound NAC transcription factor, GhNAC157, had the strongest transcriptional activity (Fig. 7a). We also performed transactivation assays in the yeast strain Y2H, with the transactivation capacity determined by X-α-Gal (5-bromo-4-chloro-3-indoxyl a-D-galactoside) activity. Almost all selected NAC proteins had transactivation activity (blue colonies), except for GhNAC280 (Fig. 7b).

Furthermore, GhNAC157 (with TM) and GhNAC158 (without TM) were selected to investigate the transactivation activity domain. Our results showed that the C-terminal activation domain (AD) of GhNAC157 and GhNAC158 exhibited transactivation activity (Fig. 7c, d), which is consistent with previous studies. In addition, we found the TM domain of GhNAC157 in the C-terminal region did not affect the activation ability (Fig. 7d).

## Discussion

Transcription factors operate as important switches of transcription networks and regulate gene expression accurately [45]. The NAC acronym is derived from *NAM* (no apical meristem), *ATAF1/2*, and *CUC2* (cup-shaped cotyledon), three genes which were initially discovered to contain a conserved NAC domain [4, 17, 18]. In cotton, we found subdomains C and D with the highly conserved and positively charged amino-acid residues, and these two subdomains might be associated with DNA binding [5]. More recent research has shown that the *NAC* genes constitute one of the largest plant-specific transcription factor families, expanding during the evolution in land plants, possibly associated with the elaborate developmental program critical to maximize plant fitness [46].

In the present study, we took advantage of the available genome data of four sequenced cotton species, and the *NAC* genes were identified located throughout these genomes. Unlike the traditional identification methods which align the conserved NAC domains, we identified *NAC* family genes based on the whole coding genes of four cotton species. Our results show that about 0.35% ~ 0.4% genes encode NAC transcription factors in the cotton genome, with the *NAC* genes representing 5.5% ~ 5.7% of all transcription factor genes. The *NAC* gene numbers which were identified in our study are different compared with previous reports, which might be caused by the difference of the reference genomes or the identification methods [36, 43, 44]. Phylogenetic and gene homology analyses discovered the evolutionary relationship between four cotton species, and the extensive homology of *NAC* genes between diploid and tetraploid cotton is consistent with a previous study that shows tetraploid cotton species (AD) is derived from an interspecific hybridization between an A-genome ancestral species, *G. herbaceum* (A1) or *G. arboreum* (A2) and a native D-genome species, *G. raimondii* (D5) or *G. gossypioides* (D6) [47]. However, the inconsistent number of homologous gene pairs indicates the independent evolution of each subgenome pairs.

There are extensive variations in gene length, predicted protein molecular weight and protein isoelectric point, whereas the gene structures are relatively conserved in the *NAC* gene family, with about 68.2% *GhNAC* genes having three exons (Fig. 2a). In particular, a high degree of similarity was detected in gene structure and predicted protein properties between homologous gene pairs. This result proves that duplicate genes originating from the progenitors can evolve independently at the same rate and show few changes [48].

Membrane-bound transcription factors (MTF) are located in the membrane itself and are activated upon receiving of specific signal, and this regulation mode is considered to be accurate, such as membrane release [10]. Membrane-bound NAC transcription factors have been extensively identified and involved in many biological processes in different plant species [9, 11, 49, 50]. In this study, we firstly identified 68 predicted membrane-bound NAC transcription factors in four cotton species and members are mainly distributed in the same subfamily, and these genes appear to be conserved during evolution. Except for the classical structures (with one α-helical TM located in the C terminal), there are some divergent forms also identified, such as two genes possessing two TM, and TM preceding the NAC domain. These forms were also identified in tomato, however, this was not found in *Arabidopsis* and rice, and the inference is that these divergent *NAC* genes have evolved functional specificity. Phylogenetic analysis showed some clades contained membrane-bound *NAC* genes derived from all of the monocots (rice) and dicots (cotton, tomato and *Arabidopsis*), suggesting that these genes probably shared a common ancestor that predated the divergence of the monocots and dicots (Additional file 4: Figure S2d).

Upland cotton (*G. hirsutum*) is a model for studying the domestication of polyploid crops. In this study, there are higher SNP densities in *GhNAC* genes in wild cotton than that in cultivated cotton, and it is suggested that the evolution of Upland cotton was accompanied by selection and domestication (Fig. 2c). There is a higher variant range of the A subgenome than of the D subgenome in wild cotton, consistent with the previous study that Upland cotton revealed asymmetric evolution between the A and D subgenomes [42]. For selective pressure analysis, we found purifying selection acted as a primary force in the evolution of NAC genes in cotton, therefore, they might retain their ancestral functions [11].

Cotton fiber is the most important natural textile material in the world, and the improvement of fiber quality is the major goal of cotton breeding. At 16 d post anthesis, the SCW thickening stage initiates and cellulose synthesis is at a high rate, and is the main constituent (> 90%) of the mature fibers [32]. Previous studies showed there are 24 and 14 *NAC* genes which exhibit higher expression in 15 DPA fibers in *G. arboreum* and *G. raimondii*, respectively [44]. Here, we identified 38 *GhNAC* genes showing high and differential expression during fiber development, with most genes preferentially expressed at the secondary cell wall deposition stage, suggesting *NAC* genes might play an important role in this process (Fig. 4c). NAC transcription factors participating in SCW formation have been well reported, and the roles of SCW-related *NAC* genes are conserved in different plant species [13, 19, 23, 51]. Phylogenetic analysis revealed some *GhNAC* genes show homology with the SCW biosynthesis related *NAC* gene *NST1* (ANAC043) of *Arabidopsis*. Combined with previous studies, we suggest that *NAC* genes regulating cotton fiber development might do so through the regulation of secondary cell wall formation. Consistent with our hypothesis, a recent study showed that a cotton *NAC* gene, *GhFSN1*, regulates fiber quality by promoting SCW biosynthesis [25]. *GhFSN1* is a fiber-related candidate gene (*GhNAC125*) in our study. Our results show that co-expression relationships exist between *GhNAC125* and genes involved plant-type secondary cell wall biogenesis, such as *GhCESA8* (CELLULOSE SYNTHASE 8, Gh_D10G0333) and *GhCOBL4* (COBRA-LIKE4, Gh_D03G0919) (Additional file 11: Table S7). We also found that the homologous gene of *GhFSN1* (*GhNAC125*), *GhNAC267*, shows low expression in fiber, and this indicates the functional differentiation of homologous gene pairs between A subgenome and D subgenome (Fig. 4d). The comprehensive analysis of *NAC* genes in fiber development will prove instructive for their function in this developmental context.

**Fig. 8:**
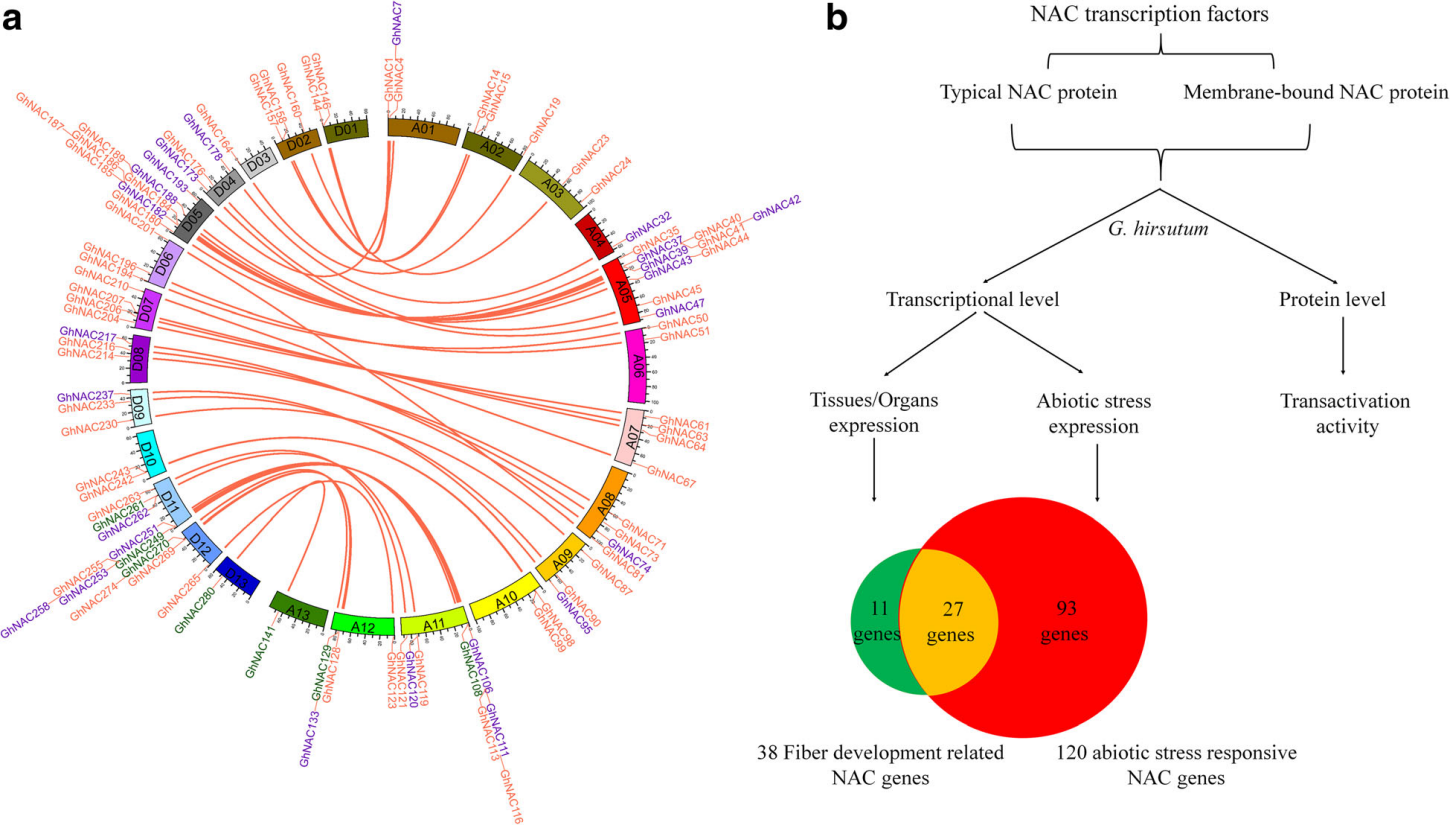
Model of gene location and functional divergence of fiber development related and stress responsive NAC genes in *G. hirsutum*. **a** Gene location of fiber development related and stress responsive NAC genes in *G. hirsutum*. There are 12 genes (*GhNAC9*, *GhNAC10*, *GhNAC33*, *GhNAC34*, *GhNAC56*, *GhNAC57*, *GhNAC59*, *GhNAC151*, *GhNAC225*, *GhNAC226*, *GhNAC275*, *GhNAC283*) have no distinct chromosome location and could not be accurately mapped. Pink fonts: stress responsive NAC genes; green fonts: fiber development related NAC genes; purple fonts: the common genes involved in these two biological processes. Lines represent homologous genes that are distributed in syntenic blocks. **b** The NAC genes were analyzed at the transcriptional (tissues/Organs expression and responsiveness to abiotic stress) and protein (transactivation activity) levels. Thirty-eight fiber development related and 120 stress responsive *GhNAC* genes were identified in this study. There are 27 genes involved in these two biological processes

Nowadays, more and more cotton cultivation areas are concentrated in marginal lands, which are threatened frequently by extreme environmental conditions, and these stresses will inevitably affect the growth, productivity, and fiber quality [52]. Much work has been performed in the past two decades to reveal the significance of NAC transcription factors in regulating various stress signaling pathways [5, 26, 53]. The study of the mechanism of cotton responses to abiotic stress has proved difficult, and so identifying valuable genes for cotton molecular breeding is necessary. Previous genomic sequencing has revealed that *NAC* genes may regulate abiotic stress tolerance in cotton [42]. Although some *NAC* genes involved in stress response have been identified, research on the biological functions and mechanisms is still in its infancy in cotton [35, 53, 54]. There are 33 *NAC* genes that respond to salt stress in *Arabidopsis*, and 40 *NAC* genes change significantly in rice under drought or salt stress [55, 56]. Here, we identified 120 (42.4%) abiotic stress-responsive *GhNAC* genes, and 29 genes showed differential expression under four stress conditions, these genes might be involved in the regulation of various stresses (Fig. 6). Most stress-responsive *GhNAC* genes changed significantly under heat and cold stresses, suggesting they might have important biological functions in response to temperature stress. It is noteworthy that the expression of some *GhNAC* genes is clustered with the stress response *NAC* genes of *Arabidopsis* and rice, and we speculate that these genes might play conserved functions in stress responses across species (Additional file 12: Fig. S5b). Previous studies partly show the validity of our results. For example, *GhATAF1* (*GhNAC173*) is highly induced by salt stress, and transgenic cotton plants overexpressing *GhATAF1* show enhanced tolerance to salt stress, by up-regulating the expression of stress-related genes and decreasing the Na^+^ content in shoots [34]. Some stress-responsive *GhNAC* genes show differential expression patterns induced by ABA treatment, and it is implied that some *GhNAC* genes could be regulated by ABA-dependent and ABA-independent pathways. In addition, 27 common *GhNAC* genes showed differential expression under stress responses and fiber development, indicating these genes may play multiple functions in cotton (Fig. 8). The stress-responsive *GhNAC* genes identified in this study could be used as promising candidates for molecular breeding to create new cotton varieties possessing good agronomic traits under adverse conditions.

## Conclusion

At present, improving the fiber quality and stress tolerance are the major goals for cotton breeding. The largest plant-specific NAC TFs are renowned for their roles in diverse developmental programs. We have comprehensively identified the *NAC* genes in four cotton species. Fiber development and stress response related *GhNAC* genes were highlighted for their expression patterns, co-expression network and transactivation (Fig. 8) This result will provide useful insights on *NAC* genes for further genetic improvement in cotton. However, it should be noted that the present analysis represents a starting point for functional studies of *GhNAC* genes on fiber development and stress response, and further experimental researches are required to expand on the findings obtained to define the biological function and molecular mechanisms of the highlighted *GhNAC* genes.

## Methods

### Identification and phylogenetic analysis of the NAC family genes in *Gossypium spp*

To identify *NAC* genes in four cotton species, we downloaded all the predicted protein sequences from CottonFGD (https://cottonfgd.org/about/download.html) [57]. *NAC* genes were identified by PlantTFDB 4.0 (http://planttfdb.cbi.pku.edu.cn/). In addition, complete amino-acid sequences were analyzed using ClustalX (ver.1.83) and MEME (http://meme-suite.org/index.html) to discover conserved motifs of NAC genes to conform our identification. The predicted molecular weight and isoelectric points of NAC proteins were calculated using the ExPASy program (http://web.expasy.org/protparam/). The membrane-bound NAC members were identified using the TMHMM server v.2.0 (http://www.cbs.dtu.dk/services/TMHMM/).

Multiple sequence alignments of the protein sequences were carried out using ClustalX (ver.1.83) with default settings. The conserved sequence logo of NAC domains (A-E) were generated using MEME (http://meme-suite.org/index.html). The complete amino acid sequences of predicted NAC proteins were used for phylogenetic analysis. Phylogenetic trees were constructed using MEGA7 software by the Neighbour-Joining method [58]. The evolutionary distances were computed using the Poisson correction method and the nodes of the trees were evaluated by boot-strap analysis with 1000 replicates.

### Gene structure, promoter, genome synteny and variation analysis of *GhNAC* genes

To analyze gene structures, the coding and genomic sequences of *GhNAC* genes were downloaded and analyzed by the online Gene Structure Display Server (GSDS2.0) program (http://gsds.cbi.pku.edu.cn/). About 1.5-kb upstream regions from the initiation codon of each *GhNAC* genes were analyzed by PlantCARE database (http://bioinformatics.psb.ugent.be/webtools/plantcare/html/) to reveal promoter cis-elements [59].

Genome synteny and variation analysis were performed as described previously [60]. The At and Dt, At and A2, Dt and D5 reciprocal aligned sequences were obtained using BLASTN (e value <1e-05). We used Circos plot to show these homologous gene pairs.

For molecular evolutionary properties, we calculated the non-synonymous (d_N_) and synonymous (d_S_) substitution rates within and between cotton species to explore the evolutionary dynamics and selection pressures [61]. The ratio d_N_/d_S_ > 1, = 1 and < 1 indicating positive (or diversifying) selection, neutral evolution, and purifying (or negative) selection, respectively. For molecular evolutionary properties analysis, the ratio of non-synonymous to synonymous for the homologous gene pairs were calculated by Maximum Likelihood (PAML) yn00 program with the GMYN method.

For variation analysis of *NAC* genes, our previous genome data from 31 wild cotton and 321 domesticated Upland cotton were used to calculate the SNP densities as previously described [60, 62].

### Expression profiles and co-expression networks analysis

The transcriptome data sets corresponding to expression abundances of TM-1 (The allotetraploid cotton *G. hirsutum* L. acc. Texas Marker-1) in different tissues and stresses from NCBI were used to analysis the expression profiles of *GhNAC* genes (https://www.ncbi.nlm.nih.gov/ sra/?term = PRJNA248163) and CottonFGD (https://cottonfgd.org/) [42, 57]. The gene expression patterns were showed by heatmap with the expression values normalized by Genesis software [63]. The expression change of *NAC* genes during fiber development were clustered by the Genesis K-means method [63].

The co-expression networks analysis of *GhNACs* involved in fiber development and stress response were performed as described previously with slight modification [60]. Pearson correlation coefficient (PCC) was used to measure the co-expression relationships between the selected *GhNAC* genes and co-expression genes. For fiber analysis, fourteen published RNA-Seq data sets were obtained, including ovule and fiber development. Weighted gene co-expression network (WGCNA) analysis was performed as described previously [64]. Genes with PCC (|PCC| ≥ 0.8 for stress, |PCC| ≥ 0.9 for fiber) were used for constructing co-expression networks. All of the co-expression networks were visualized by Cytoscape software (3.4.0). The co-expression genes were annotated based on functional gene ontology using KOBAS 3.0 [65].

### Plant materials and treatments

*Gossypium hirsutum* cv. YZ1 were cultivated in Hoagland solution in 28 °C culture room with 16 h light/8 h dark photoperiod cycle conditions. Four-week-old seedlings were treated with Hoagland solution containing 200 mM NaCl, 15% PEG, and 0.5 μM ABA, respectively. The leaves and roots were collected at different time intervals, immersed in liquid nitrogen immediately and then frozen at − 80 °C for later use. To test the expression of selected genes in different tissues/organs, the methods for tissue samples collection were performed as previously described [66].

### qRT-PCR analysis

qRT-PCR analysis was performed as described previously with little modification [66]. High-quality RNA was reverse transcribed to cDNA using SuperScript III Reverse Transcriptase in accordance with the manufacturer’s instructions (Cat. No.18080–093, Invitrogen). qRT-PCR experiments were performed using an ABI Prism 7500 system (Applied Biosystems) and the comparative Ct (2^-△△Ct^) method was used to calculate gene expression levels. *GhUBQ7* (GenBank accession No.DQ116441) was used as the internal control. Gene-specific primers for qRT-PCR were designed according to the cDNA sequences using Primer Premier 5.0 software and synthesized commercially (Genscript Bioscience). The primers used for qRT-PCR are listed in Additional file 14: Table S8.

### Transcriptional activation analysis

The transactivation activation ability of NAC proteins was performed by protoplast transient expression system as previously described [66]. Reporter construct contained five copies of the GAL4 binding site in tandem and a minimal TATA region of the CaMV 35S promoter, the firefly gene for luciferase (LUC). Selected *NAC* genes were cloned into the vector GAL4DB and transformed into *Escherichia coli* TOP 10. After culturing, the plasmids were extracted and transferred to protoplast derived from cotton embryogenic calluses using PEG-calcium transformation method as described previously [67]. A dual-luciferase assay was used to detect the LUC and Renilla LUC (Promega, Cat. no. E1910).

For yeast transactivation assay, a bait protein is expressed as a fusion to the Gal4 DNA-binding domain (DNA-BD), the gene coding sequences were cloned respectively into the pGBKT7 vector to achieve this goal. Here, the full-length coding sequences of *GhNAC* genes and the truncated forms (*GhNAC157-BD*, *GhNAC157-AD*, *GhNAC157△TM*, *GhNAC158-BD*, *GhNAC158-AD*) were cloned respectively into the pGBKT7 vector. These vectors were transformed into Y2H gold strains according to the instruction manual (Cat. No.630489, Clontech), and transactivation activity was determined by the growth on SD medium without Trp and added X-α-Gal (SD-Trp + X-α-Gal). The photograph was taken after 36 h incubation. The primers used in the transactivation assay are listed in Additional file 14: Table S8.

## Additional files

Additional file 1: **Table S1.** Protein sequences and nomenclature of cotton NAC genes. (XLSX 166 kb)

Additional file 2: **Figure S1.** Statistical analysis of sequence length, molecular weight and isoelectric point for NAC protein in cotton. (TIF 478 kb)

Additional file 3: **Table S2.** Basic characteristic of NAC genes in cotton. (XLSX 54 kb)

Additional file 4: **Figure S2.** The deviant structures of membrane-bound *NAC* genes in cotton. (a) GhNAC157 had a TM at C terminal. (b) GaNAC111 possessed two TM at C terminal. (c) NAC genes with TM preceded the conserved NAC domain. (d) Phylogenetic analysis of membrane-bound NAC transcription factors between cotton and other plant species. Phylogenetic tree was made by MEGA7 software through the Neighbour-Joining method. *G. hirsutum* (red square), *G. barbadense* (green square), *G. raimondii* (blue triangle), *G. arboreum* (magenta triangle), *Arabidopsis* (blue hollow square), *Oryza sativa* (magenta hollow triangle), *Solanum lycopersicum* (black hollow triangle). (TIF 3906 kb)

Additional file 5: **Figure S3.** The inter-genomic (At and Dt) synteny analysis of NAC genes. Lines represent homologous genes that are distributed in syntenic blocks. The ‘t’ indicates tetraploid. (TIF 4880 kb)

Additional file 6: **Table S3.** The chromosome location of *NAC* genes in *G. hirsutum*. (XLSX 25 kb)

Additional file 7: **Table S4.** The homologous gene pairs were identified in cotton. (XLSX 14 kb)

Additional file 8: **Table S5.** Cis-element analysis of *NAC* genes in *G. hirsutum*. (XLSX 34 kb)

Additional file 9: **Figure S4.** The expression patterns of *NAC* genes. (a) The expression patterns of *GhNAC* genes in different tissues/organs. (b) The expression patterns for homologous genes of the 38 highly expressed *GhNAC* genes during 10 and 20 DPA fiber development in four cotton species. (TIF 4910 kb)

Additional file 10: **Table S6.** The transcriptome data for fiber development and stress responsive *GhNAC* genes. (XLSX 102 kb)

Additional file 11: **Table S7.** Co-expression genes with selected *NAC* genes involved in fiber development and stress response. (XLSX 31 kb)

Additional file 12: **Figure S5.** The expression analysis of stress-related *GhNAC* genes. (a) The expression patterns of *GhNAC* genes under abiotic stress. (b) Phylogenetic relationship of GhNAC proteins with previously reported stress related NAC proteins in *Arabidopsis* and rice. ANAC019 (AT1G52890), ANAC055 (AT3G15500), RD26 (AT4G27410), ANAC002 (AT1G01720), SNAC1 (Os03g0815100), SNAC2 (XP_015620920). (c) GO enrichment analysis of co-expression genes. (TIF 2882 kb)

Additional file 13: **Figure S6.** The expression of selected *GhNAC* genes under ABA (0.5 μΜ) treatment. The *GhUBQ7* (GenBank accession number: DQ116441) was used as the internal control. Bars represent means ± standard error (*n* = 3). (TIF 503 kb)

Additional file 14: **Table S8.** Primer sequences were used in this study. (XLSX 11 kb)

## Abbreviations

A2: A-genome species
ABA: Abscisic acid
At: A subgenome, ‘t’ denoting tetraploid
BD: DNA-binding domains
D5: D-genome species
DEG: Differentially expressed genes
d_N_: Non-synonymous
DPA: Days post anthesis
d_S_: Synonymous
Dt: D subgenome
HSE: Heat stress response element
LUC: Luciferase
MTF: Membrane-bound transcription factors
NAC: NAM, ATAF, and CUC
PEG: Polyethylene glycol
SCW: Secondary cell wall
SNP: Single nucleotide polymorphism
TM: Transmembrane motif
TR: Transcriptional regulatory regions
X-α-Gal: 5-bromo-4-chloro-3-indoxyl a-D-galactoside
