# Supplementary figures and images for: Comprehensive analysis of NAC transcription factors uncovers their roles during fiber development and stress response in cotton
Source: BMC Plant Biol. 2018 Jul 24;18:150. doi: 10.1186/s12870-018-1367-5 (PMC6057059; doi:10.1186/s12870-018-1367-5)

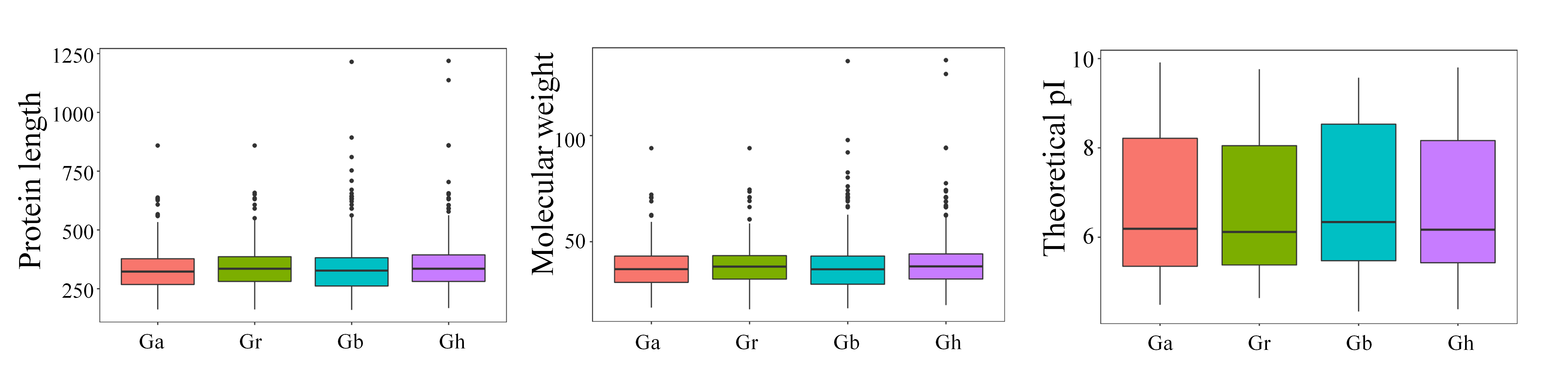

Supplement: Supplementary file 2 — Figure S1. Statistical analysis of sequence length, molecular weight and isoelectric point for NAC protein in cotton. (TIF 478 kb) [file 12870_2018_1367_MOESM2_ESM.tif]

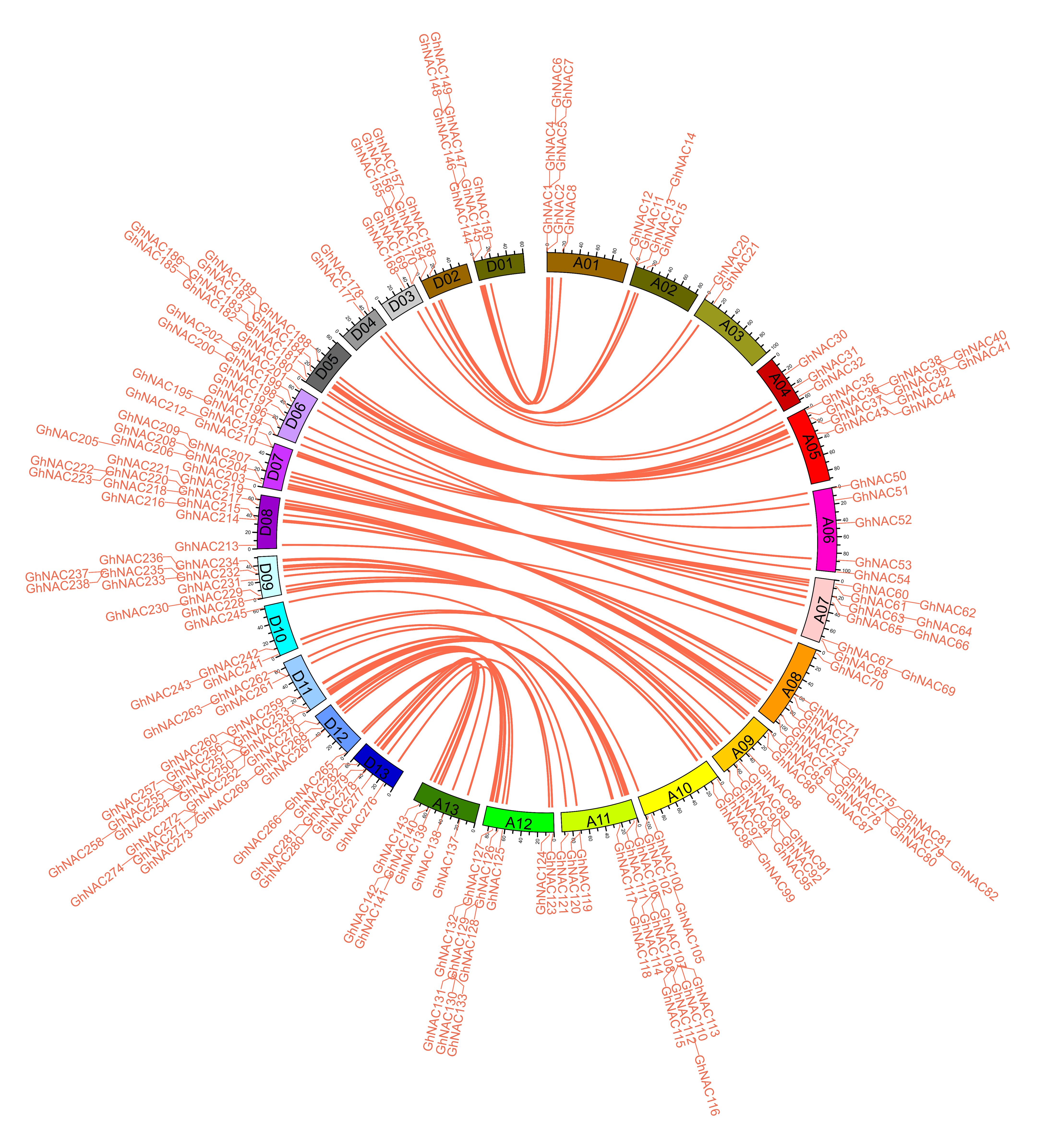

Supplement: Supplementary file 5 — Figure S3. The inter-genomic (At and Dt) synteny analysis of NAC genes. Lines represent homologous genes that are distributed in syntenic blocks. The ‘t’ indicates tetraploid. (TIF 4880 kb) [file 12870_2018_1367_MOESM5_ESM.tif]

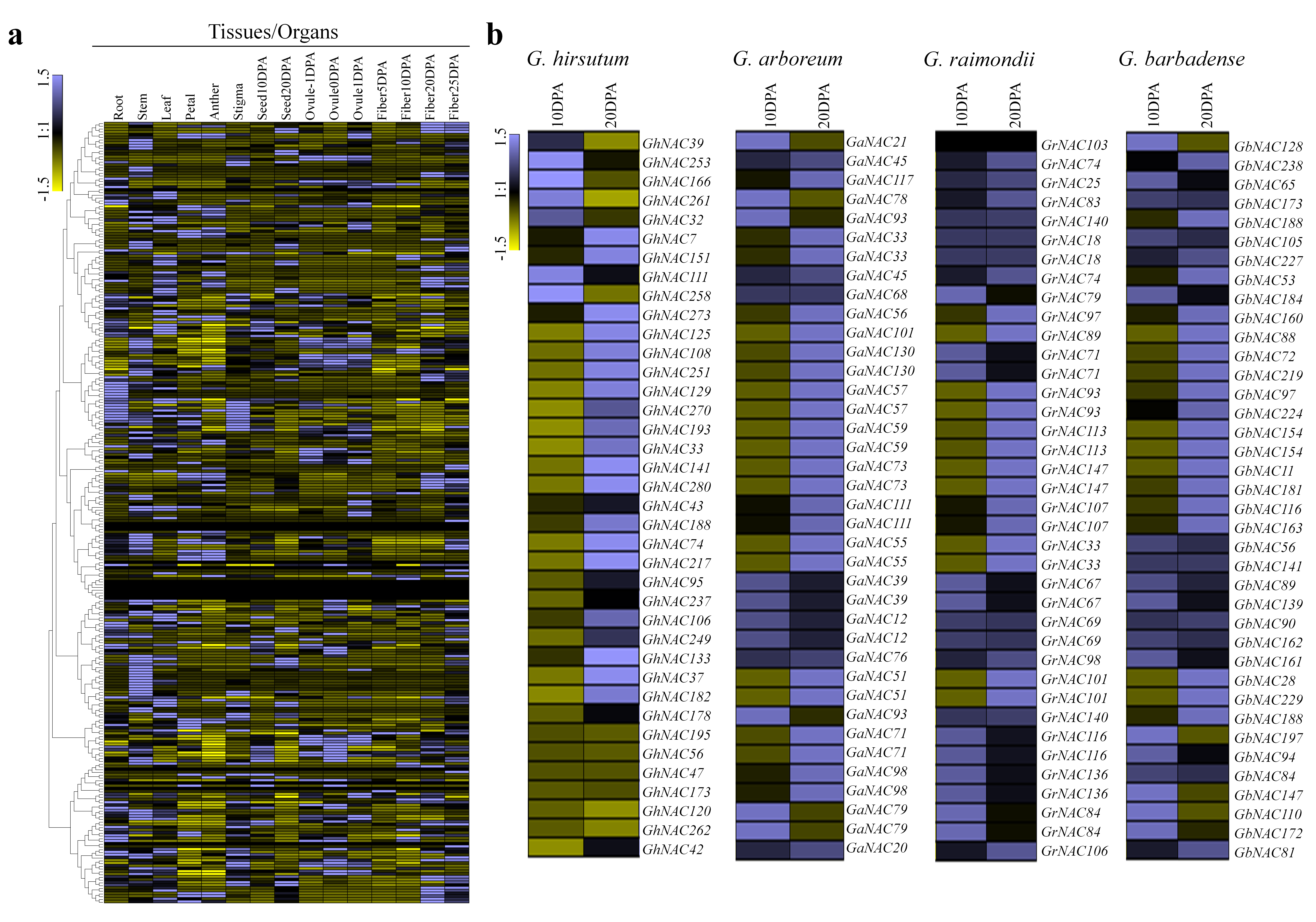

Supplement: Supplementary file 9 — Figure S4. The expression patterns of NAC genes. (a) The expression patterns of GhNAC genes in different tissues/organs. (b) The expression patterns for homologous genes of the 38 highly expressed GhNAC genes during 10 and 20 DPA fiber development in four cotton species. (TIF 4910 kb) [file 12870_2018_1367_MOESM9_ESM.tif]

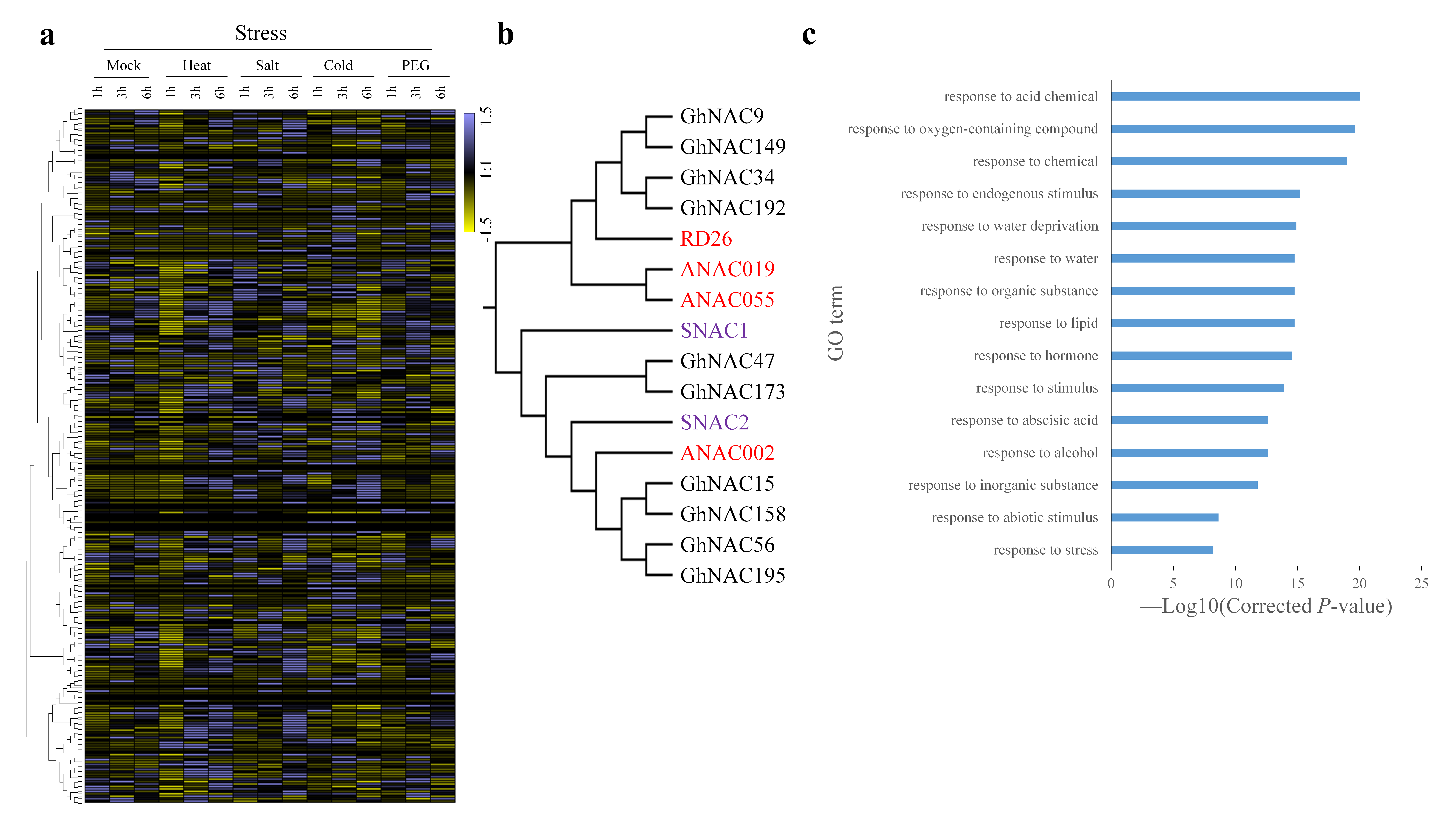

Supplement: Supplementary file 12 — Figure S5. The expression analysis of stress-related GhNAC genes. (a) The expression patterns of GhNAC genes under abiotic stress. (b) Phylogenetic relationship of GhNAC proteins with previously reported stress related NAC proteins in Arabidopsis and rice. ANAC019 (AT1G52890), ANAC055 (AT3G15500), RD26 (AT4G27410), ANAC002 (AT1G01720), SNAC1 (Os03g0815100), SNAC2 (XP_015620920). (c) GO enrichment analysis of co-expression genes. (TIF 2882 kb) [file 12870_2018_1367_MOESM12_ESM.tif]

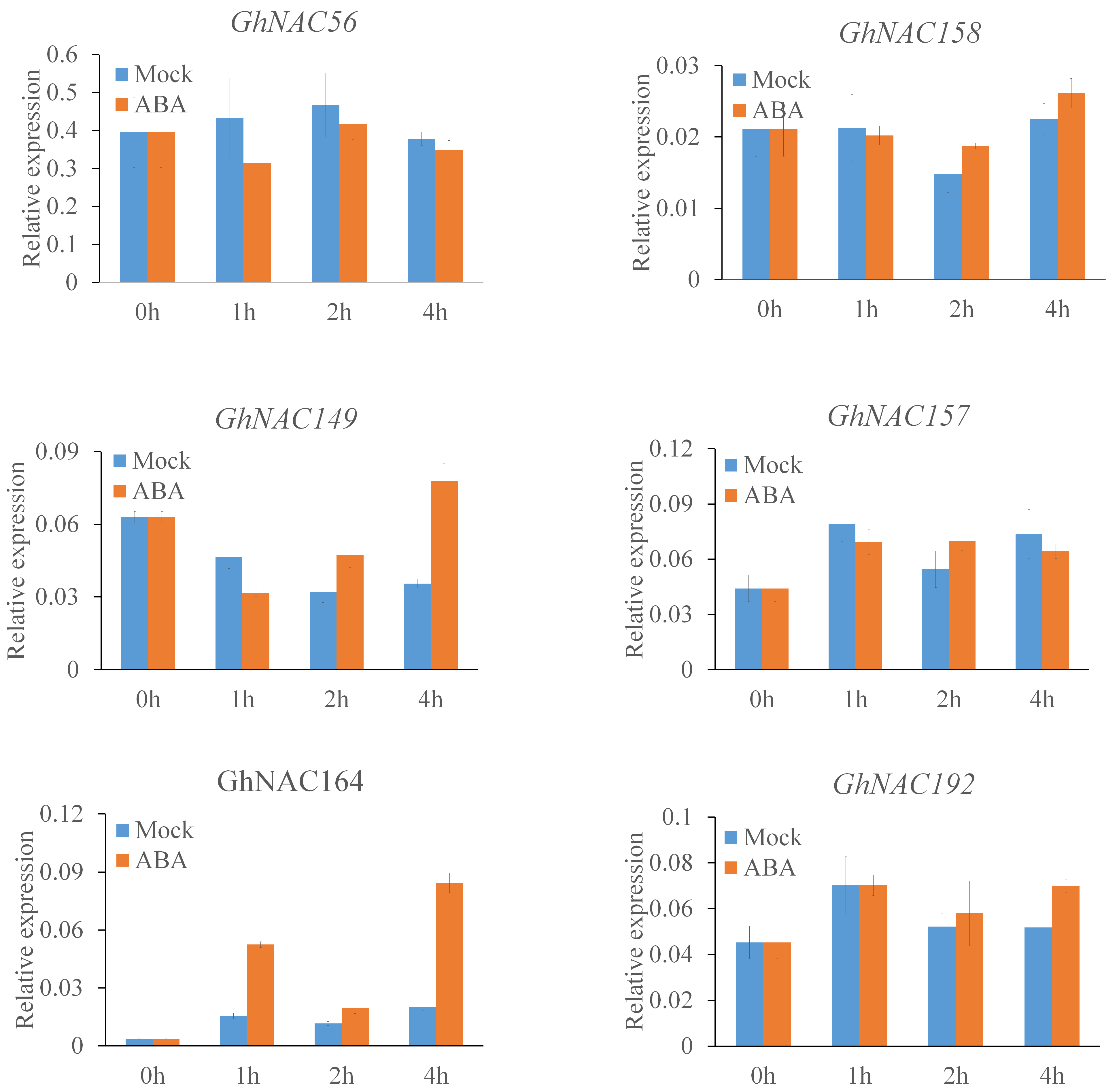

Supplement: Supplementary file 13 — Figure S6. The expression of selected GhNAC genes under ABA (0.5 μΜ) treatment. The GhUBQ7 (GenBank accession number: DQ116441) was used as the internal control. Bars represent means ± standard error (n = 3). (TIF 503 kb) [file 12870_2018_1367_MOESM13_ESM.tif]
